# Evaluation of the Rosemary Extract Effect on the Properties of Polylactic Acid-Based Materials

**DOI:** 10.3390/ma11101825

**Published:** 2018-09-25

**Authors:** Raluca Nicoleta Darie-Niţă, Cornelia Vasile, Elena Stoleru, Daniela Pamfil, Traian Zaharescu, Liliana Tarţău, Niţă Tudorachi, Mihai Adrian Brebu, Gina Mihaela Pricope, Raluca Petronela Dumitriu, Karol Leluk

**Affiliations:** 1 Department of Physical Chemistry of Polymers, “Petru Poni” Institute of Macromolecular Chemistry, 41A Gr. Ghica Voda Alley, 700487 Iasi, Romania; darier@icmpp.ro (R.N.D.-N.); pamfil.daniela@icmpp.ro (D.P.); ntudor@icmpp.ro (N.T.); bmihai@icmpp.ro (M.A.B.); rdumi@icmpp.ro (R.P.D.); 2 National Institute for Electrical Engineering (INCDIE ICPE CA), 313 Splaiul Unirii, P.O. Box 149, 030138 Bucharest, Romania; traian_zaharescu@yahoo.com; 3 Grigore T. Popa University of Medicine and Pharmacy Iasi, 16 University Street, 700115 Iasi, Romania; lylytartau@yahoo.com; 4 Veterinary and Food Safety Laboratory, Department of Food Safety, 700115 Iasi, Romania; ginacornelia@yahoo.com; 5 Institute of Environmental Protection Engineering, Wroclaw University of Technology, Plac Grunwaldzki 9, 50-377 Wroclaw, Poland; kleluk@yahoo.com

**Keywords:** powdered rosemary ethanolic extract, poly(lactic acid), bioactive food packaging, biomaterials

## Abstract

New multifunctional materials containing additives derived from natural resources as powdered rosemary ethanolic extract were obtained by melt mixing and processed in good conditions without degradation and loss of additives. Incorporation of powdered rosemary ethanolic extract (R) into poly(lactic acid) (PLA) improved elongation at break, rheological properties, antibacterial and antioxidant activities, in addition to the biocompatibility. The good accordance between results of the chemiluminescence method and radical scavenging activity determination by chemical method evidenced the increased thermoxidative stability of the PLA biocomposites with respect to neat PLA, with R acting as an antioxidant. PLA/R biocomposites also showed low permeability to gases and migration rates of the bioactive compounds and could be considered as high-performance materials for food packaging. In vitro biocompatibility based on the determination of surface properties demonstrated a good hydrophilicity, better spreading and division of fibroblasts, and increased platelet cohesion. The implantation of PLA/R pellets, was proven to possess a good in vivo biocompatibility, and resulted in similar changes in blood parameters and biochemical responses with the control group, suggesting that these PLA-based materials demonstrate very desirable properties as potential biomaterials, useful in human medicine for tissue engineering, wound management, orthopedic devices, scaffolds, drug delivery systems, etc. Therefore, PLA/R-based materials show promising properties for applications both in food packaging and as bioactive biomaterials.

## 1. Introduction

The use of natural additives is gaining increasing interest in the development of new multifunctional materials as a key for new active materials strategies. Different natural essential oils have been proposed for incorporation into the polymer matrices to improve the functionality as well as the products (food and pharmaceuticals) quality and safety.

The action of natural additives is essential in reducing or even eliminating some of the main spoilage causes, such as rancidity, color loss/change, active compounds losses, dehydration, microbial proliferation, senescence, gas build-up, and off-odors, etc. [1].

Nature offers several solutions to obtain appropriate antioxidants with higher activity than those which are synthesized. Ethanolic extraction is a method by which the highest quantity of phenolic compounds can be extracted from plant leaves. Isolated compounds, as well as crude ethanol extract, showed antibacterial activities against four Gram-negative bacteria strains, *Escherichia coli*, *Pseudomonas aeruginosa*, *Klebsiella pneumoniae*, and *Enterobacter aerogenes* [2], in addition to antioxidant activity because the content in phenolic compounds is preserved [3].

**Figure 1:**
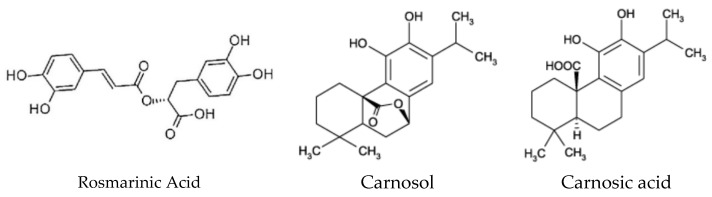
Chemical structure of the major antioxidative compounds in rosemary extracts.

The use of extracts from rosemary (*Rosmarinus officinalis*) as food preservatives is well established [4,5]. A broad range of beneficial health effects can be attributed to rosemary, such as antidepressant, antihypertensive, antiproliferative, antibacterial, antiatherogenic, hypocholesterolemic, hepatoprotective, and anti-obesity properties. The biological properties of rosemary are attributed to the contribution of its different bioactive compounds belonging mainly to the classes of phenolic acids, flavonoids, diterpenoids, and triterpenes. Rosemary samples have the highest levels of flavonoids and other compounds such as carnosol, rosmaridiphenol, rosmadial, rosmarinic acid, and carnosic acid [6]. It was found to be very efficient to protect food against lipid oxidation [7] and is known as rosemary active packaging [8]. Antioxidative efficiency is imparted by at least 20 specific phenols, the most effective compounds are carnosol, rosmarinic acid, and carnosic acid, followed by caffeic acid, rosmanol, rosmadial, genkwanin, and cirsimaritin. Rosemary extracts derived from *Rosmarinus officinalis* L. contain several compounds which have been shown to possess antimicrobial, antioxidative, anti-inflammatory, antiviral, and anti-tumor functions. Literature reports either rosmarinic acid, an ester of caffeic acid and 3,4-dihydroxyphenyllactic acid or/and the phenolic diterpenes carnosol and carnosic acid as the principal antioxidative components of the rosemary extract (Figure 1) [9,10].

Almost 90% of the rosemary leaf extract’s antioxidant activity can be attributed to carnosol and carnosic acid [11].

Knowing that oxidative stress plays a determinant role in the pathogenesis of liver diseases, at this moment the antioxidant products from natural sources are being increasingly used to treat various pathological liver conditions. Due to its antioxidant and antimicrobial properties, rosemary essential oil (REO) is already largely used in the food industry as a preservative, while additionally possessing other health benefits. In addition to free radical scavenging activity, the REO mediates the hepatoprotective effects by activating the physiological defense mechanisms. Rašković et al. [12] have found 29 chemical compounds of a selected REO, and the main ones identified were 1,8-cineole (43.77%), camphor (12.53%), and α-pinene (11.51%). The essential oil they tested has exerted hepatoprotective effects on rats with carbon tetrachloride-induced acute liver damage, at doses of 5 mg/kg and 10 mg/kg, by diminishing aspartate transaminase (AST) and alanine aminotransferase (ALT) activities by up to two-fold and preventing lipid peroxidation in liver homogenates. Moreover, pre-treatment with the tested essential oil for seven days has significantly reversed the activities of antioxidant enzymes (such as catalase, peroxidase, glutathione peroxidase, and glutathione reductase) in liver homogenates, mainly using a dose of 10 mg/kg.

Polylactic acid (PLA) is one of the well-known biodegradable, biocompatible, non-toxic, and eco-friendly polyesters. The low cost and attractive materials properties of PLA would open many applications. Despite the great advantages of PLA, it presents poor toughness, thus its use in obtaining materials that require high deformation capabilities is limited. There are several ways to improve PLA’s processability, flexibility, and ductility, such as: blending with polymers, copolymerization with other monomers, plasticization using biocompatible plasticizer, or incorporation of filler materials [13]. The most promising method to increase PLA flexibility for film manufacturing as well as to improve the compatibility between PLA and other additives in the blend has been proven to be blending with low-molecular-weight polymers, which act as plasticizers [14]. An efficient plasticizer for PLA must reduce the glass transition temperature and the crystallinity, not migrate and show miscibility with the matrix, and possess a low volatility and a lack of toxicity [15]. Depending on the targeted application, there are various types of plasticizers for PLA, but in view of obtaining sustainable materials, mostly eco-friendly plasticizers are used today [16]. Literature shows possible plasticizers used for PLA processing, such as, oligomeric lactic acid, l-lactide, poly(ethylene glycol) (PEG), epoxidized soybean oil (USE), citrate esters, glycerol, and glucose monoesters [14,17]. Among these, the most common and suitable plasticizer for PLA has been reported to be low-molecular-weight PEG due to its miscibility, biodegradability, glass transition temperature (*T*_g_) reduction, and approval for food-contactable materials [13,17]. The major characteristics of PEG are its biocompatibility, water solubility, and low cost that recommend its use in biomaterials composition [18].

PLA and its composites are currently being extensively studied to obtain degradable food packaging and are also widely used in human medicine for tissue engineering, wound management, orthopedic devices, biomedical and clinical applications in scaffolds, bone fixation devices such as screws and plates, surgical suture and meshes, medical implants, drug delivery systems, etc. [19,20,21,22].

As natural additives are too volatile to be directly used in the processing technologies, a viable alternative is to incorporate these compounds into materials as natural active additives with the possibility to be released from the material of interest and to reduce their volatility. The release rate of active substances such as antioxidants from a packaging material can be evaluated through migration studies, usually performed using food simulants and conditions specified in the European food packaging regulations [23,24]. As it is desirable to place the samples in contact with the food simulant in the worst foreseeable conditions of time and temperature pertaining to actual use, and as increasing the temperature accelerates the migration, the conditions for the release studies were 40 °C for a minimum of ten days with a 50% aqueous ethanol solution used as a food simulant. This is known as a modified D1 food simulant, which is assigned as a conventional simulant selected for foods with lipophilic character, oil-in-water emulsion character, hydrophilic foods containing relevant amounts of organic ingredients, and for hydrophilic alcohol-containing foods with over 20% alcohol content. Generally, food simulant D1 can be considered to be the most severe aqueous food simulant for hydrophilic non-acidic foods [23]. Since ethanol is commonly used as a food simulant, the 50% aqueous ethanol solution was chosen to perform these tests in order to comply with the latest approved directives on migration testing (EU EC. No. 10/2011) [23]. Through ethanol sorption in the PLA matrix, voids or swelling of the film can occur, facilitating penetration within PLA chains and promoting the migration of the active substances [25,26].

In this paper antimicrobial and antioxidant activity was offered to the PLA by mixing with ethanolic rosemary extract as a powder. This was obtained by alcoholic extraction of the rosemary leaves followed by precipitation [27].

## 2. Experimental

### 2.1. Materials

Polylactic acid (PLA) obtained from renewable resources from NatureWorks LLC (trade name: PLA 2002D, Minnetonka, MN, USA) is a transparent material with a melt flow index of 5–7 g/10 min (conditions 210 °C/2.16 kg) and a content of 96% l-lactide and 4% isomer D. Average molecular weight determined by GPC was 4475 kDa. It has a density of 1.25 g/cm^3^, melting point of 152 °C, glass transition temperature of 58 °C. The crystallinity depends on the isomer content and thermal history. Water permeability at 25 °C was 172 g/m^2^_*_day and percentage of biodegradation/mineralization is 100% [16].

Poly(ethylene glycol) (PEG) BioUltra 4000 purchased from Sigma-Aldrich (St. Louis, MO, USA) was used as a plasticizer.

Powdered rosemary ethanolic extract (R) was obtained following a previously reported procedure by the solvent extraction method in a Soxhlet unit [27] involving the alcoholic extraction of the rosemary leaves followed by precipitation. Fresh rosemary leaves were collected from local farms, dried at ambient temperature and subsequently milled, cleaned, and powdered in a grinder (Laboratory of Radiation Chemistry, INCDIE-ICPE CA. Bucharest, Romania). The vegetal material was then refluxed in 2 L of ethanol at 65–75 °C for 10 h during continuous extraction in the Soxhlet apparatus (Laboratory of Radiation Chemistry, INCDIE-ICPE CA. Bucharest, Romania). The extract in ethanol solution was subjected to precipitation induced by the addition of water as non-solvent and a solid phase was finally filtered, washed with acetone, dried in air, and further dried under vacuum at ambient temperature. A greenish-yellow fine powder was obtained and stored in desiccators to avoid the absorption of moisture.

All other reagents were of analytical grade purity.

The composition of the rosemary ethanolic extracts was detailed and studied because of the importance of its biological activity in various applications. As previously mentioned in the introduction, the rosemary extracts, especially the ones derived from the leaves, are usually herbal products used as natural antioxidant and flavoring agents in food processing and cosmetics. In the present study, the phenolic and flavonoids contents have been determined according to the following methods.

#### 2.1.1. Determination of Total Phenolic Content

Total phenols content was determined using Folin-Ciocalteu’s reagent (FC) method as presented in the work of Scalbert et al. [28]. Powdered rosemary extract (0.01 g) was added to 10 mL methanol and then 0.1 mL of methanolic solution was diluted with 0.4 mL of double distilled H_2_O. FC/H_2_O solution (1:10 *v*/*v*) (1 mL) was added to 0.5 mL diluted methanol/R extract and left for 10 min at 25 °C after a strong mixing. A 2 mL solution of 15% sodium carbonate (Na_2_CO_3_·10H_2_O) was added, and after 1 h of incubation, the absorbance was read at 740 nm with a Cary 60 UV-Vis spectrophotometer (Agilent Technologies, Santa Clara, CA, USA) against a freshly prepared blank sample. The blank sample contained 0.1 mL methanol without powdered rosemary ethanolic extract, 0.4 mL of water, 1 mL FC reagent solution, and 2 mL of Na_2_CO_3_·10H_2_O solution. Solutions of gallic acid (used as standard) in methanol of different concentrations (0.01–1 mg/mL) were prepared and based on the measured absorbance at 740 nm, the calibration curve was drawn. The obtained calibration curve was then used for the determination of the total phenolics content expressed as mgGAE/g dw (mg of gallic acid equivalent per g of dry weight). The determination was made in triplicate and an average value was obtained. The resulted total phenolic content was of 112.5 mg GAE/g dw.

#### 2.1.2. Determination of Total Flavonoids Content

Total flavonoid content was determined by the aluminum chloride colorimetric assay [29]. A quantity of 1 mL of extract (1 mg/mL powdered rosemary ethanolic extract) or standard Quercetin (of different concentrations) was added to 4 mL of H_2_O. Then, at different time intervals the following solutions were added consecutively: 0.30 mL of 5% NaNO_2_; 0.3 mL of 10% AlCl_3_ after 5 min; and 2 mL of 1 M NaOH after another 5 min. At the end, the volume was made up to 10 mL with H_2_O, mixed, and absorbance was measured against the blank sample at 510 nm. The total content of flavonoids was expressed as mg Quercetin Equivalents (QE)/g dw. The measurement was made in triplicate and an average value was reported. Thus, a total flavonoids content of 261.5 (mg QE/g dw) was found.

The obtained values both for phenolic and flavonoids content of the powdered ethanolic rosemary extract are close to those found in the literature, which explain, in part, its good antioxidant properties [30].

### 2.2. PLA-Based Blends Processing

PLA-based blends were prepared using different amounts of powdered ethanolic rosemary extract (R) by incorporation into a PLA matrix in the melt state. Before mixing, drying of the additives and PLA was done in a vacuum oven (Binder, Tuttingen, Germany) for 6 h at a temperature of 80 °C. The compounding was performed for 10 min, at 175 °C, and 60 rpm, by means of a Brabender mixer (30EHT, Duisburg, Germany). A Carver press (Wabash MPI, IN, USA) and special parameters for compression molding (175 °C, or 165 °C for PLA/R blends, pre-pressing for 3 min at 50 atm and a pressing for 2 min at 150 atm) were used to obtain specimens for different analyses. Both films (thickness of ~0.15 mm), as well as sheets (thickness of ~1 mm), were prepared for different analyses that required specific thickness. Sheets were used for mechanical (tensile) and rheological tests (1 mm distance between parallel plates) while films were used for spectral characterization, permeability tests, etc. The compositions of the prepared systems are shown in Table 1.

### 2.3. Investigation Methods

#### 2.3.1. Processing Behavior

Processing behavior was evaluated by analysis of processing characteristics following the torque-time curves registered during blending on a Brabender mixer.

#### 2.3.2. Scanning Electron Microscopy (SEM)

The samples were fixed on copper supports for the SEM investigation and the surface of the films was examined as such, without metal coating. An Environmental Scanning Electron Microscope (ESEM) type Quanta 200 Instrument (FEI Company, Hillsboro, TX, USA) was used, operating at 25 kV with secondary electrons in low vacuum mode (LFD detector).

#### 2.3.3. ATR–FTIR Spectroscopy

A Bruker VERTEX 70 spectrometer (Ettlingen, Germany) was used to record the Attenuated Total Reflectance-Fourier Transformed Infrared ATR-FTIR spectra by a 4 cm^−1^ resolution. Both the spectra of samples as well as background were recorded in the wavenumber interval of 4000–600 cm^−1^, and the OPUS program was used for the processing of spectra.

#### 2.3.4. Stress-Strain Measurements

Tensile measurements were performed at room temperature on dumbbell-shaped samples, by means of an Instron Single Column machine (3345, Norwood, MA, USA), according to EN ISO 527-2/2012. The load cell was of 1 kN, the loading speed was of 10 mm/min, and the gauge length was 40 mm. Young’s modulus, tensile strength at break, and strain at break have been evaluated. 

#### 2.3.5. Dynamic Rheology

An Anton Paar rheometer (MCR301, Graz, Austria) was used to study the rheological properties of the PLA/R composites. Oscillatory frequency sweeps were performed using a geometry with a parallel plate of 25 mm in diameter, at 175 °C, and a strain of 10% (linear viscoelasticity region) in the range between 0.05 to 500 rad/s.

#### 2.3.6. Differential Scanning Calorimetry (DSC)

Thermal analysis of PLA/R composites was performed under a nitrogen atmosphere by means of a TA Instruments Q20 Dynamic Scanning Calorimeter (New Castle, DE, USA). Each sample of ~10 mg was cooled down to 0 °C and heated up to +250 °C, which was below the glass transition and above the melting temperature of the materials. After the first heating run, each sample was kept for 2 min and then cooled down to 0 °C with a cooling rate of 5 °C/min and reheated up to 250 °C with a heating rate of 10 °C/min. As PLA is a semicrystalline polymer with a low crystallinity, the cooling rate must be slower than the heating rate in order to increase the crystallinity. An empty crucible was used as a reference. The parameters achieved through the DSC experiments were the glass transition temperature, cold crystallization, as well as the melting temperature and crystallinity. The degree of crystallinity of the investigated samples was obtained by dividing the melting enthalpy of the sample by *ΔH_m_* = 93.7 J/g [31], which is equilibrium enthalpy of PLA sample with 100% crystallinity (PLA 100%).

#### 2.3.7. Thermogravimetry–Infrared Spectroscopy/Mass Spectrometry (TG-FTIR/MS) Coupled Analysis (TG-FTIR/MS)

The thermal behavior of the samples and evolved gases analyses were performed using a TG-FTIR/MS system. The system is equipped with a thermobalance STA 449F1 Jupiter (Netzsch, Germany), spectrophotometer FTIR model Vertex-70 Bruker, (Ettlingen, Germany) and mass spectrometer QMS 403C Aёolos (Netzsch, Germany). The thermogravimetric analyzer was calibrated for temperature and sensitivity using the melting point of some standard metals (Hg, In, Sn, Bi, Al) from −38.5 °C to 800 °C. The temperature reproducibility was ±2 °C. The heating program was between 30–600 °C, with a 10 °C min^−1^ heating rate and nitrogen as a carrier gas with a flow rate of 50 mL min^−1^. The samples (7–8 mg) were heated in an open Al_2_O_3_ crucible, and Al_2_O_3_ was used as the reference material. The gases released during the thermal degradation were transferred by two isothermal transition lines to the FTIR and mass spectrometer. The transfer line to the FTIR spectrophotometer is heated at 190 °C, the gases are introduced in TGA-IR external modulus (gas cell V = 8.7 mL) and the spectra are recorded on a 600–4000 cm^−1^ domain with a resolution of 4 cm^−1^. The acquisition of FTIR spectra in 3D was done with OPUS 6.5 software (Ettlingen, Germany) that measures from the start and synchronizes across all the apparatus. On the other hand, the transfer line to the mass spectrometer is heated at 300 °C and the volatile degradation products are directly transferred to an electron impact ion source of 70 eV. The data acquisition was achieved with Aeolos^®^ 7.0 software (Netzsch, Germany), in the scan bar graph mode on the range *m*/*z* = 1–300.

#### 2.3.8. Chemiluminescence

Chemiluminescence (CL) spectra were recorded on small-weight samples (max. 5 mg) by means of a LUMIPOL 3 unit (SAS, Bratislava, Slovakia) instrument as non-isothermal dependencies of the recorded intensity on the temperature of samples. The temperature interval used for measurements started from room temperature until 250 °C, and a low error (±0.5 °C) was associated with measured temperatures. CL measurements were performed in air under static conditions using different heating rates, namely 2, 3.5, 5, and 10 °C/min. The intensity values of CL were normalized to sample mass for their reliable comparison. 

#### 2.3.9. Antioxidant Activity Evaluation

The ABTS•^+^ (2,2′-azino-bis(3-ethylbenzothiazoline-6-sulfonic acid) diammonium salt radical cation scavenging assay can be used to assess the antioxidant activity of both hydrophilic and hydrophobic compounds. To obtain the ABTS•^+^ radical cation, 2.5 mL of 7 mM ABTS is mixed with 2.5 mL 14.7 mM potassium persulphate (KPS) aqueous solutions and the mixture is left in the dark, at room temperature, for 16 h. By the direct reaction between ABTS and KPS, the ABTS•^+^ radical cation is generated, which is a blue-green chromophore, that can undergo a reduction reaction in the presence of an antioxidant. This reaction determines a loss of absorbance at 750 nm. Using Equation (1), the antioxidant capacity was calculated and the resulting values were expressed as percentage inhibition.

(1) $$Inhibition(\%)=\left[\frac{A_{control}-A_{sample}}{A_{control}}\times 100\right]$$

In the case of polymeric composites, the reaction mixture consisted of adding different volumes (mL) of the reaction mixture and 2 mL of ABTS radical solution. The reaction mixture was obtained by placing 80 mg of sample in 5 mL ethanol and stirred for 24 h at room temperature (20 °C).

#### 2.3.10. Antimicrobial Activity

The antimicrobial activity against *Bacillus cereus*, *Salmonella typhymurium*, and *Escherichia coli*, included four experimental steps as follows. The first stage was the sterilization of samples in an autoclave at 110 °C and 0.5 bars for 20 min. The second step consisted in the American Type Culture Collection (ATCC) culture bacteria contamination where the preparation of ATCC cultures was done by seeding of the pre-enrichment medium and incubation for 24 h at 37 °C; then, the colonies were counted in 0.1 mL culture by selective culture medium separation; and 0.1 mL bacterial culture ATCC was seeded using a sterile swab samples surface. The third step consisted in the incubation of samples contaminated with the ATCC for 24 h at 25 °C in the dark, in sterilized glass Petri dishes, repeated for another 24 h incubation. The last step involved the identifying of target germs. The following standardized methods of bacteriology procedures were used, according to standards in force: SR ISO 16649—*E. coli*; ISO 7932:2004 ISO 21871:2006(en)—*Bacillus cereus*, SR EN ISO 6579—*Salmonella* spp.

#### 2.3.11. Gas Permeability

The gas transmission rates of CO_2_ and O_2_ were recorded by means of a Lyssy L100-5000 (Systech Instruments Ltd., Johnsburg, IL, USA) manometric gas permeability tester (~4 h to equilibrium for CO_2_ and ~7 h to equilibrium for O_2_). Specimens with 108 mm × 108 mm × 0.2 mm dimensions were used. Four determinations were made for each sample and the average value was reported.

#### 2.3.12. Migration Study

The migration of the active components belonging to the powdered rosemary ethanolic extract from the different polymeric PLA-based films was investigated by a total immersion migration test (EC, 1997) [32] using 50% aqueous ethanolic solution as a food simulant assigned for fatty foods which frequently undergo oxidation [33]. The release studies were performed at 40 °C for a minimum of 10 days by keeping the samples in an oven, and the selected testing conditions simulate storage at ambient temperature for an unlimited duration. The migration tests were performed with pieces of films of about 1 cm^2^ in 5 mL of simulant. A blank test for the simulant and each type of control sample was carried out previously. 

Aliquots of ~1 mL withdrawn from the release medium at predetermined time intervals were analyzed at 285 nm by means of a Cary 60 UV-VIS spectrophotometer (Agilent Technologies, Santa Clara, CA, USA) by scanning from 200 to 600 nm. Samples were run in quartz cuvettes with a 1 mm path length.

The active components’ concentrations were calculated based on the calibration curve previously determined at 285 nm for the main components of rosemary extract, sincefor rosmarinic acid the λ_max_ values are reported in literature as 254, 290, and 328 nm, and for carnosol and carnosic acid at 283 and 246 nm [34,35]. The chosen λ_max_ value can be attributed to overlapped maxima corresponding to rosemary extract active ingredients since all of them contain the aromatic double bond. 

The corresponding release curves were represented as time-dependent plots of the cumulative percentage of active component released. 

In order to evaluate the migration kinetic parameters and to establish the release mechanism involved, the release data were fitted first to the Korsmeyer-Peppas model using the Equation (2) [36,37]:

*M_t_*/*M_∞_*= k·*t^n^* (2)

The migration data were also fitted to the Higuchi kinetic model, using the Equation (3):

*M_t_*/*M_∞_* = k_H_·*t*^1/2^ (3)

where *M_t_*/*M_∞_* represents the fraction of bioactive compound (s) released at time *t*, *n* is the release exponent and k and k_H_ are the release rate constants for each model considered.

The power law release exponent n describes the release mechanism from a thin polymer sample: a value of *n* = 0.5 corresponds to a Fickian diffusion mechanism, 0.5 < *n* < 1 to non-Fickian/anomalous transport, *n* = 1 to Case II transport, and *n* > 1 to super case II transport [36,37].

#### 2.3.13. Biocompatibility Evaluation

##### In Vitro Biocompatibility Evaluation—Contact Angle (CA) and Surface Free Energy (SFE).

By the sessile drop method, the contact angle (CA) of the polymeric sample surfaces was measured using a CAM-200 instrument from KSV (Helsinki, Finland) at room temperature and a controlled humidity. A 1 μL drop of water was placed on the film’s surface and after 10 s the static contact angle was recorded. To evaluate the wettability at least ten contact angle CA measurements were realized in different locations on the surface and the obtained average values were used further. For more details on the method see references [38,39]. To determine the surface free energy (SFE) components, the CA at equilibrium between the film surface and three pure liquids (in addition to water, methylene iodide, and formamide were used, as-purchased at maximum obtainable purity) were measured by fitting the drop profile using the Young-Laplace equation [40,41,42,43]. The acid/base (LW/AB) approach of van Oss and Good, see Equation (4) [44,45], was used to calculate the total SFE and its components, namely the dispersive component, also named the Lifshitz–van der Waals interaction, ($\gamma_{sv}^{LW}$) and polar Lewis acid-base interactions ($\gamma_{sv}^{AB}$), respectively, see Equation (5). The acid-base interactions are subdivided into electron donor $\gamma_{sv}^{-}$ (Lewis base) and electron acceptor $\gamma_{sv}^{+}$ (Lewis acid) parts, see Equations (4)–(6). The subscripts “*lv*” and “*sv*” denote the liquid-vapor and surface-vapor interfaces.

(4) $$(1+\cos\theta )\gamma_{lv}=2\left(\sqrt{\gamma_{sv}^{LW}\gamma_{lv}^{LW}}+\sqrt{\gamma_{sv}^{+}\gamma_{lv}^{-}}+\sqrt{\gamma_{sv}^{-}\gamma_{lv}^{+}}\right)$$

(5) $$\gamma_{sv}^{AB}=2\sqrt{\gamma_{sv}^{+}\gamma_{sv}^{-}}$$

(6) $$\gamma_{sv}^{TOT}=\gamma_{sv}^{LW}+\gamma_{sv}^{AB}$$

where *θ* is the contact angle, $\gamma_{sv}$ is the liquid’s total surface tension, and $\gamma_{lv}^{LW}$ and $\gamma_{sv}^{LW}$ are the apolar (dispersive) Lifshitz–van der Waals components of the liquid and the solid, respectively, whereas $\gamma_{sv}^{+}\gamma_{lv}^{-}$ and $\gamma_{sv}^{-}\gamma_{lv}^{+}$ are the Lewis acid–base contributions of either the solid or the liquid phase as indicated by the subscripts. To determine the total surface free energy of the solid material ($\gamma_{sv}^{TOT}$) a system based on Equation (4) must be used. To solve the resulting systems of equations it is necessary to use at least three test liquids with known $\gamma_{lv}$, $\gamma_{lv}^{LW}$, $\gamma_{lv}^{-}$, and $\gamma_{lv}^{+}$, see Table 2 [45,46,47,48].

The manner in which the surface of the materials interacts with the constituents of blood, such as platelets and red blood cells, determines if the tested materials are blood compatible. Evaluating the surface/interfacial free energy of a material can represent an in vitro method to determine biocompatibility. Equation (7) was used to establish if the obtained materials present blood compatibility, where *W_s/rbc_* and *W_s/p_* denote the red blood cells and platelets work spreading [49]. When a biomaterial is exposed to blood it can cause the adhesion of blood cells onto surface, and the extent of this adhesion will determine the life of the implanted biomaterials. By such cells adherence to biomaterial surfaces the coagulation and immunological cascades could be activated [50].

(7) $$W_{s}=W_{a}-W_{c}=2\left(\sqrt{\gamma_{sv}^{LW}\gamma_{lv}^{LW}}+\sqrt{\gamma_{sv}^{+}\gamma_{lv}^{-}}+\sqrt{\gamma_{sv}^{-}\gamma_{lv}^{+}}\right)-2\gamma_{lv}$$

where *W_s_*—work of spreading (the negative free energy associated with spreading liquid over the solid surface); *W_a_*—work of adhesion (defined as the work required separating the liquid and solid phases) and *W_c_*—work of cohesion (defined as the work required separating a liquid into two parts) [41].

##### In Vivo Biological Evaluation

White male Wistar rats (200–250 g) were used in the experiment. The animals were housed under a standard laboratory environment (relative humidity 55–65%, chamber temperature 23.0 ± 2.0 °C and 12 h of light: Dark sequence (lights on at 6:00 a.m.) and fed with a specific diet and water ad libitum, excluding the time of the investigations. Before the assessment, the animals were positioned on a raised wire mesh, under a clear Plexiglass container and allowed 2 h to familiarize themselves to the testing room.

Rats were randomly assigned into eight groups of six animals each according to the following protocol: Group 1: Control (C)—distilled water; Group 2: R—powdered rosemary ethanolic extract; Group 3: PLA; Group 4: PLA/0.25R; Group 5: PLA/0.50R; Group 6: PLA/0.75R; Group 7: PLA/PEG; and Group 8: PLA/PEG/0.5R.

In the first day of the investigations, under general anesthesia (with ketamine 50 mg/kg body weight and xylazine 10 mg/kg body weight), the samples (weighing 62 mg) were placed subcutaneously, in one side in the rat’s dorsal region, after a small incision, and subsequently sutured. Cotton pellets, of 62 mg, impregnated with distilled water (0.3 mL) and respectively with R solution (0.3 mL), were subcutaneously inserted into control animals. Pellets and the implanted samples behaved like foreign materials generating a subacute inflammatory response.

The in vivo biocompatibility of PLA-based materials containing rosemary ethanolic extract was evaluated by estimating the influences on the hematological and serum biochemical tests and on certain immune system parameters [51,52].

After certain time intervals (24 h and 7 days) following the implantation of PLA-based materials containing rosemary ethanolic extract, 0.3 mL of blood samples were collected from retro-orbital plexus and the following parameters were determined: Blood count, aspartate transaminase (AST), alanine aminotransferase (ALT), and lactic dehydrogenase (LDH) activity, as well as, serum urea and creatinine levels [53].

At the same time in the experiment, the serum complement activity, and the phagocytic capacity of peripheral neutrophils (Nitro Blue Toluene—NBT test) were also established [54]. These parameters belong to specific tests used to quantify the influence of pharmacologic agents on the immune defense capacity of laboratory animals [55].

The data were expressed as mean +/− standard deviation (S.D.) and processed using SPSS variant 17.0 for Windows 10, to estimate the differences between the control group and the groups receiving the investigated substances. The values of coefficient p (probability) below 0.05 compared with those of the control group were considered to be statistically significant.

Ethics statement: The experimental research protocol was approved by the local Animal Ethics Committee of the “Grigore T. Popa” University of Medicine and Pharmacy, Iași, Romania, in strict observance of the international ethical regulations on laboratory animal work (AVMA Guidelines on Euthanasia, 2007). Biocompatibility tests were performed according to the “Grigore T. Popa” University of Medicine and Pharmacy guidelines for the handling and use of experimental animals and in accordance with the recommendations and policies of the International Association for the Study of Pain [56].

Each animal was used only once and the length of the experiments was kept as short as possible. For ethical considerations, all the animals were euthanized at the end of the experiment [57].

## 3. Results and Discussion

Films and sheets resulting from the melt mixing process are transparent and homogeneous and at high R concentration show a little yellowing.

### 3.1. Processing Behavior

The torque-time curves were registered during blending in a Brabender mixer in order to evaluate the processing behavior by analyzing torque values at different mixing times. Melt processing characteristics of PLA and its blends—Table 3—are not changed by the incorporation of the powdered rosemary ethanolic extract.

A sharp change in the melt mixing behavior was recorded in the presence of a plasticizer. PEG improved the melt flow, the materials being easily processed as all torque characteristics decreased. PEG favors the processing of polymers due to the increased chain mobility, therefore improving macromolecular movement. This effect was previously observed by other authors for various types of blends containing PLA and PEG, for example in PLA/PEG/organoclays nanocomposites [13] or PLA/PHB blends [58]. No oxidation occurred during processing.

### 3.2. SEM Results

**Figure 2:**
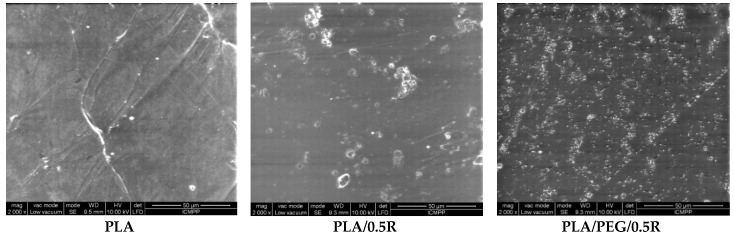
SEM images of PLA, PLA/0.5R and PLA/PEG/0.5R. PEG is defined as poly(ethylene glycol) and R is defined as rosemary ethanolic extract.

SEM images, presented in Figure 2, show a homogeneous distribution of the rosemary powder in the PEG plasticized PLA samples without agglomeration, which happens in the absence of the plasticizer.

**Figure 3:**
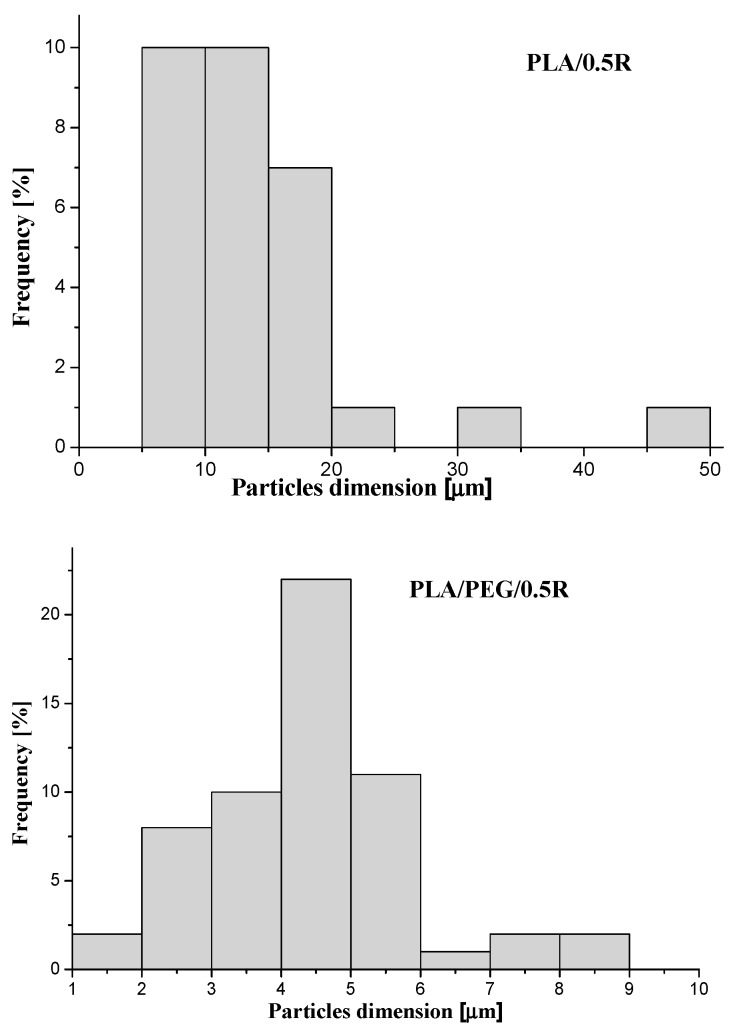
The distribution of particle dimensions determined from SEM images.

From SEM images, the histograms of the distribution of the particle dimensions were evaluated and are shown in Figure 3. For the PLA/0.5R sample, an average dimension of the particles of 14.2 µm was found while for PLA/PEG/0.5R this was approximately 4.1 µm. Therefore, the incorporation of PEG into the PLA-based composites improves the R distribution, with the sample being more homogenous and the particles dimension histogram being narrower.

### 3.3. ATR-FTIR Data

**Figure 4:**
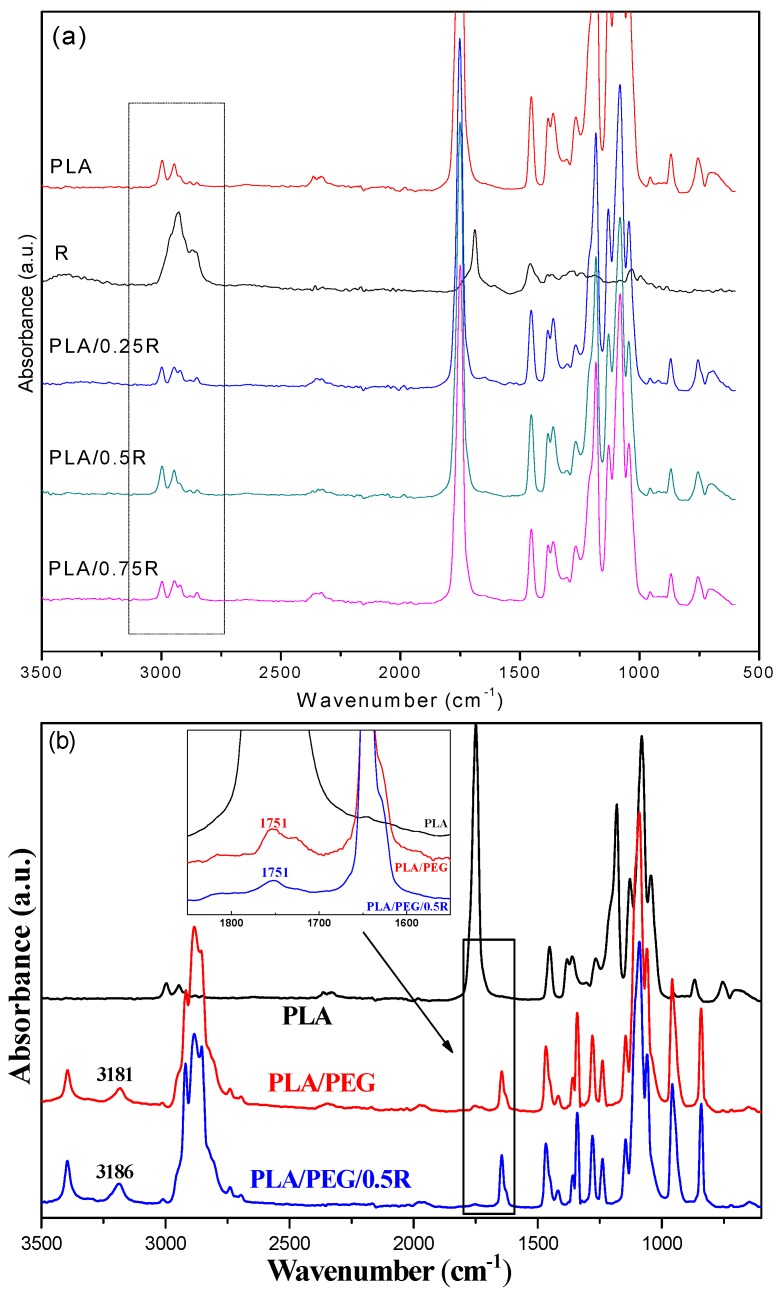
FTIR spectra of the neat PLA and the PLA/rosemary blends (**a**) and PLA/PEG/R (**b**); insert spectrum highlights details of different spectral regions.

The ATR-FTIR spectra of the PLA prior to and after loading with powdered rosemary ethanolic extract are presented in Figure 4. The IR spectrum of the PLA presents a strong band at 1749.3 cm^−1^ due to C=O stretching of the carbonyl group, while the bending vibration of this group appears at 1267.1 cm^−1^. The bands at 867.9 cm^−1^ and 754.1 cm^−1^ are assigned to the amorphous and respectively crystalline phases of PLA. The IR bands at 2995.2 and 2945.1 cm^−1^ are assigned to the CH stretching as *ν*_as_ CH_3_ and *ν*_s_ CH_3_ modes.

The components of powdered rosemary ethanolic extract have different groups in their structures (such as COOH, C–O, phenolic) with corresponding absorption bands in the region of 1750–1600 cm^−1^. The R sample exhibits a relatively sharp band with a maximum at 1692 cm^−1^ corresponding to C–O vibrations, which are also present in the samples loaded with powdered rosemary ethanolic extract. The rosemary constituents are mainly aromatic compounds and the band appearing at 1026 cm^−1^ is attributed to the deformation vibration of C–H bonds from aromatic rings [59].

The shape of the bands in the 2500–3500 cm^−1^ region, see the highlighted region of Figure 4a, are specific for each sample, as they are a coupling of the bands of PLA and R with insignificant shifts, while in the case of the systems containing plasticized PLA the bands’ shift indicates some interactions between components due to the better distribution of the components. These bands are associated with the stretching modes of C–H overlapped with the –OH stretch from alcohols, carbonyl in aldehydes, and carboxyl groups [60].

### 3.4. Mechanical Properties

**Figure 5:**
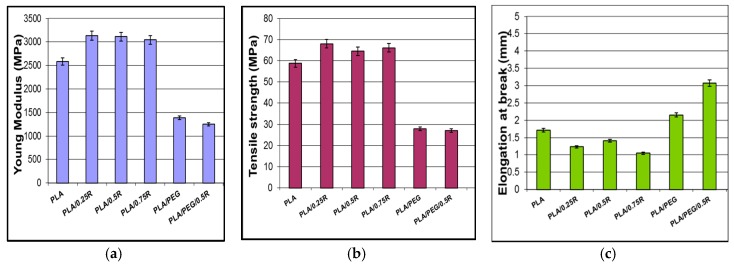
Mechanical properties of the PLA/R and PEG-plasticized PLA/R: (**a**) Young’s modulus; (**b**) tensile strength; (**c**) elongation at beak.

The Young’s modulus and tensile strength increase after incorporation of R into PLA and decrease in plasticized PLA, while as is expected, the elongation at break increased and is two times higher for plasticized systems containing R– as revealed in Figure 5. An increase in the elongation at break and a decrease in the tensile strength for PLA/PEG4000 blends were previously reported [61], with ~7% elongation at break for the PLA/PEG4000 blend [62]. The mechanical properties are kept within satisfactory limits with improved elasticity for plasticized samples in the presence of R. Examples in the literature present a lack of systems containing PLA/rosemary, mostly using essential oils; therefore, it is difficult to compare results with different types of materials processed in other conditions. A PLA-based compound comprising rosemary essential oil showed a slightly decreased elongation at break by 2.8% [63].

### 3.5. Rheological Behavior

**Figure 6:**
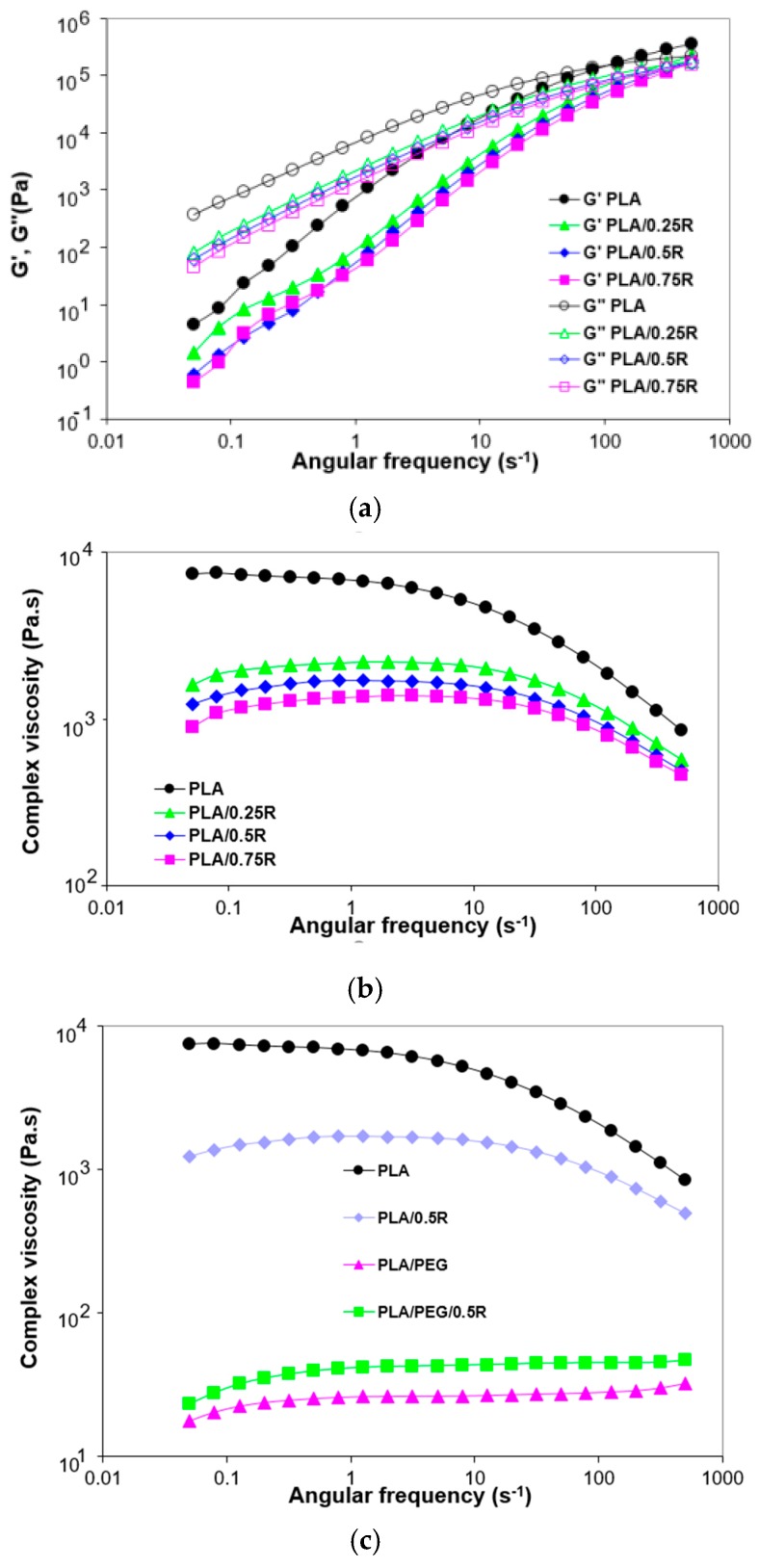
Angular frequency dependence of the storage modulus and loss modulus (**a**) and complex viscosity (**b**,**c**) for PLA, PLA/R and PLA/PEG/R systems.

The incorporation of the powdered rosemary ethanolic extract in the PLA films led to a decrease of all studied rheological parameters: Storage modulus (*G′*), loss modulus (*G*″), and complex viscosity, as presented in Figure 6a,b. A predominantly viscous behavior (*G″* > *G′*) can be noticed both for PLA and for the PLA-based composites. The loss modulus dependence on the deformation frequency presents the same decreasing trend for the composites investigated with the differences becoming more pronounced at low frequencies. The results obtained are supported by those found by other authors comparing various formulations with that containing rosemary; the flow curve of the rosemary-containing formulation is the lowest one [64].

The incorporated R amount maintains the decreasing order of moduli and viscosity, the highest content (0.75%) causes a drastic decrease of complex viscosity in the melt state as was also observed from the torque-time curves recorded during melt processing.

The lowest value is obtained for plasticized PLA, see Figure 6c, while the addition of R to plasticized PLA resulted in a small increase of the complex viscosity.

**Figure 7:**
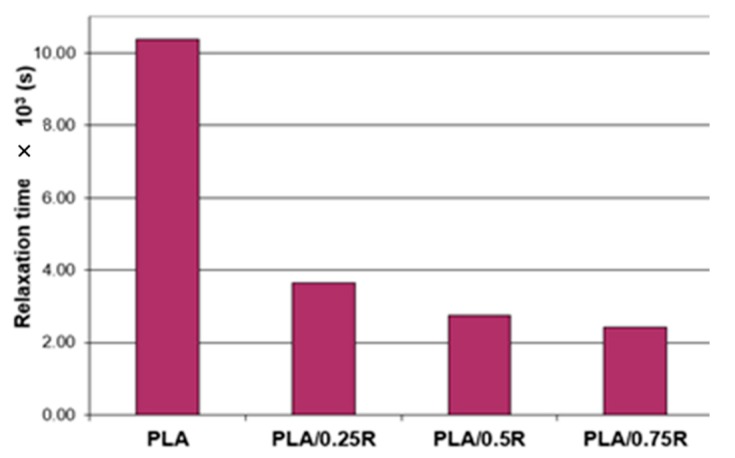
Relaxation time vs. sample composition.

As shown in Table 4, the crossover frequency (*ω*i) and the crossover modulus (corresponding to *G*″ = *G*′) were obtained at higher oscillation frequencies for the blends with an increasing R content incorporated. The relaxation time (*θ*)—calculated as *θ* = 1/ωi and the crossover modulus values obtained for PLA and its blends are plotted in Figure 7. For the PLA/0.75R blend, the relaxation time decreased by more than 23% compared with neat PLA, showing that less time was required for reorientation of the entangled chains in these cases, as the rosemary powder induced relaxation and thus flexibility of the chains.

### 3.6. DSC Results

**Figure 8:**
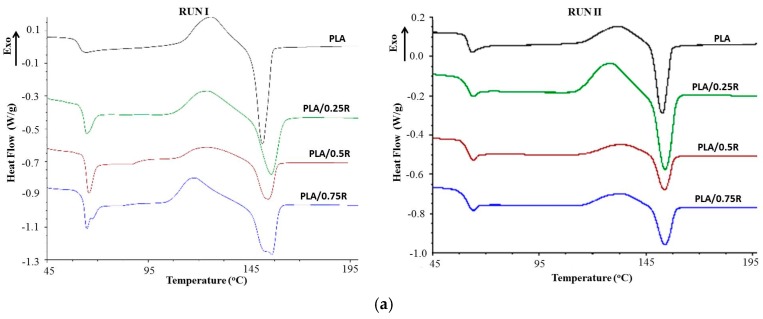

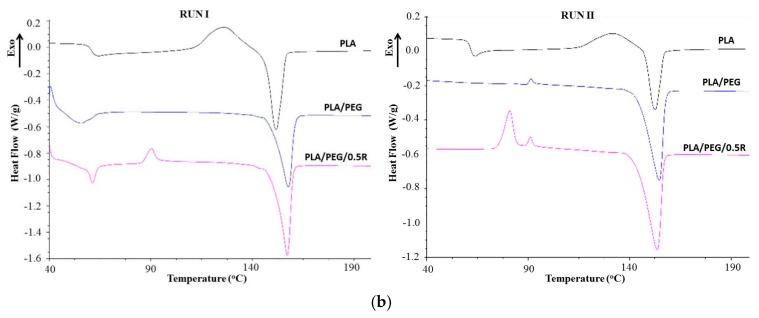
Differential scanning calorimetry (DSC) curves of the PLA and PLA/R (**a**) and plasticized PLA/R systems (**b**).

The DSC curves of the PLA/R and PLA/PEG/R systems recorded both in run I and II are shown in Figure 8 and values of the thermal properties are summarized in Table 5. In the DSC curves, all particular transitions of PLA, namely, glass transition temperature (*T_g_*), cold crystallization temperature (*T_cc_*), and melting temperature (*T_m_*) are evidenced, which show some differences between the studied systems.

There is a strong dependence of the thermal properties of the powdered rosemary ethanolic extract containing samples on their particle distribution into the PLA matrix. In the binary systems, because of the agglomeration of particles, the majority of the properties decrease or remain unchanged while in the binary and ternary blend containing plasticizer the thermal characteristics are changed, eventually resulting in an increased degree of crystallization. The thermal characteristics recorded in the first run are a little different from those recorded in the second run, but the variation with sample composition is almost similar. *T_g_* increases with R content and decreases as is expected when plasticizer is incorporated, and increases again for the ternary system PLA/PEG/0.5R. The *T_cc_* recorded in both runs decreases for all studied systems, especially for PLA/PEG/0.5R. The decrease is less significant for values corresponding to the second run. The melting temperature *T_m_*, increases in all cases; the most important increase was observed for plasticized PLA systems. As the plasticized systems are highly ordered the melting peak is very pronounced, especially in the second run because the plasticizer favors arrangement of the chains during cooling and pre-melting processes, increasing the degree of crystallinity. The results are in accordance with those found in other papers [16,17]. The enthalpy of the cold crystallization (*ΔH_cc_*) is approximately constant for binary PLA/R systems and is very small for plasticized PLA systems, while the enthalpy of melting process (*ΔH_m_*) decreases by R incorporation and increases in the cases of systems containing plasticizers because of the good distribution of particles and better mobility of the chains which favors the three-dimensional arrangements. As a result, the crystallization degree (*X_cr_)* decreases by R incorporation and increases for plasticized PLA systems. 

### 3.7. TG-FTIR/MS Data

**Figure 9:**
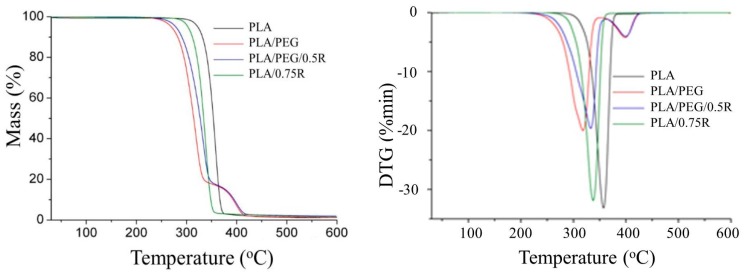
TG/DTG curves of the PLA, PLA/R, and PLA/PEG/R systems.

As can be seen from the TG/DTG curves in Figure 9, the PLA and PLA/R samples decompose in a single step observed from 310–380 °C, evolving more than 98% as volatile products, see Table 6. Under an inert atmosphere, a lower thermal stability of the samples containing R was found. The plasticized PLA systems decomposed in two steps, the first one as the main step occurring in the temperature range of 280–370 °C with a mass loss of 82–83%, indicating a significant influence of the plasticizer on the thermal decomposition. The second step was assigned to the decomposition of the residue formed in the first step. It takes place in the temperature region of 365–430 °C with a mass loss of 15–16%. Comparing the onset and *T*_10_ decomposition temperature of the PLA/PEG and PLA/PEG/0.5R, results indicated that the incorporation of R led to an increased thermal stability of the system.

**Figure 10:**
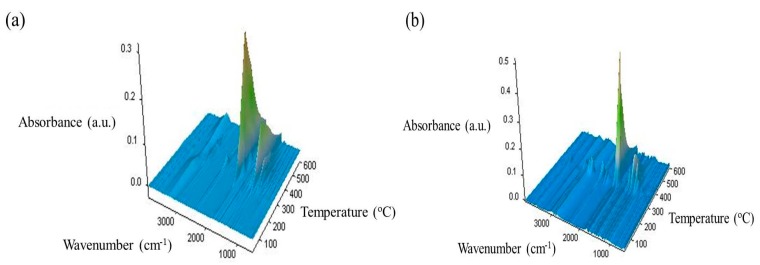
3D-FTIR spectra of the volatile decomposition products resulting from the thermal degradation of PLA (**a**) and PLA/0.75R (**b**) samples.

As it concerns the type of decomposition products, some information has been obtained by coupling TG with FTIR/MS spectroscopies. Looking at the results from the data in Figure 10 showing the 3D FTIR spectra, the change in the thermal decomposition products after incorporation of R is evident.

The FTIR spectra show differences both in the intensity of the bands and also in the PLA/0.75R system. The number of bands is increased or important shifts in the PLA bands are observed due to the possible interaction between components of the system.

**Figure 11:**
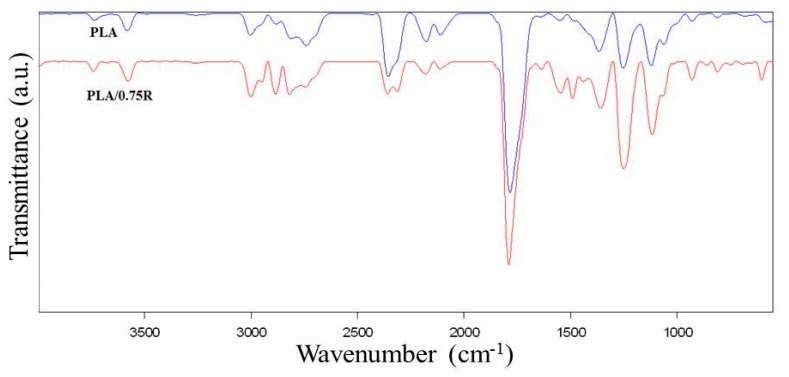
2D FTIR spectra of the thermal degradation products resulting from PLA and PLA/0.75R.

The new bands in the 2D spectra of the thermal degradation products resulting from PLA/0.75R residue, see Figure 11, have been identified as: 2949.8 cm^−1^ (C–H stretching of CH_2_ and CH_3_); 2313.7, 1631.9 cm^−1^ (C=O, C–N and COO^−^ stretching); 1490.4 cm^−1^ (aromatic domain and N–H bending, C–O stretching vibration (amide), and C–C stretching from phenyl groups, COO– stretching, CH_2_ bending; 861; 747.9 and 602.5 cm^−1^ (C–H out-of-plane bending vibration from isoprenoids, etc.) [65].

The important shifts of the following bands are found from 2812 cm^−1^ in PLA to 2820 cm^−1^ in PLA/0.75R; 2841 cm^−1^ to 2845 cm^−1^; 1785 cm^−1^ to 1791 cm^−1^; 1367.7 cm^−1^ to 1359 cm^−1^; 1122 to 1117.2 cm^−1^ and 680.6 to 691.6 cm^−1^. In the FTIR spectrum of degradation products, the following new bands and shifts of the bands appeared at approximately: 1736, 1673, 1366, 1243, 862, and 750 cm^−1^ are found. These bands probably correspond to the fragments resulting from α—pinene and 1,8-cineole products which may evolve from rosemary ethanolic extract. These products appear together with those resulting from the PLA matrix.

**Figure 12:**
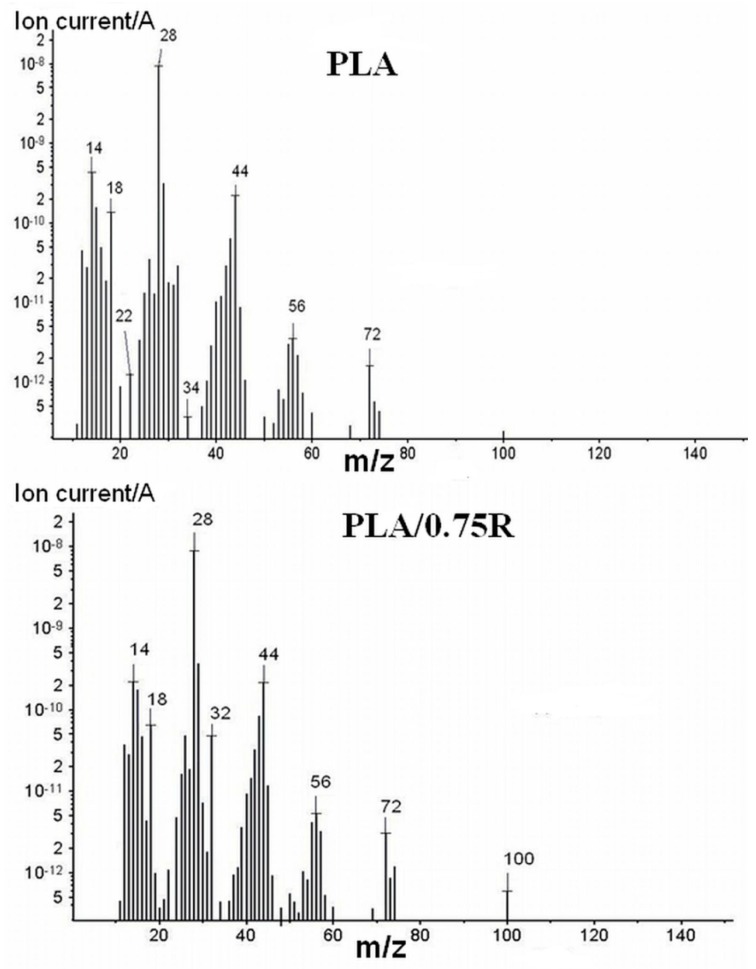
MS spectra of the thermal degradation products resulted from PLA and PLA/0.75R.

At *T* > 420 °C all samples are almost totally degraded according to results from the TG/DTG curves, with a residual amount of ~2%. Therefore, by FTIR/MS some of the thermal degradation products from the resulting mixtures at the final temperature are identified. Comparing these results with those obtained from the MS in Figure 12 indicated different products resulted by thermal degradation of PLA and its blends containing alcoholic extract of rosemary.

The pyrolysis of neat PLA results in the production of a large number of cyclic oligomers through the random degradation process. In accordance with the literature data, the main fragments of lactide meso-form or DL-form [66,67] are identified for *m*/z = 32, 43, 45 and 56. PLA, in general, shows a dominant series of signals with *m/z* = 56 + (n × 72) in which n assumed values of 1, 2, 3, and 4. Acetaldehyde (*m*/*z* 15, 26 and 43), 2,3-pentadione, and acrylic acid were also identified. Further decomposition products were H_2_O, CO_2_, and hydrocarbons (*m*/*z* of 18, 44, 12–17, etc., respectively) [68]. The presence of the plasticizer led to very complex spectra.

### 3.8. Chemiluminescence

The stability of PLA is related to the formation of lactide structures [69]. Under gamma irradiation poly(l-lactic acid), the random scission of molecular chains takes place, which results in the sharp decrease in the numerical average molecular weight [70]. Moreover, due to the exposure to gamma radiation, several free radicals centered on carbon atoms are formed which release volatile products or restore polymeric configurations. The radiochemical behavior is the mirror of radiation processing during sterilization and grafting, when the weaker sites, α-methyl positions and branching places, become the main sources of degradation initiators [71].

**Figure 13:**
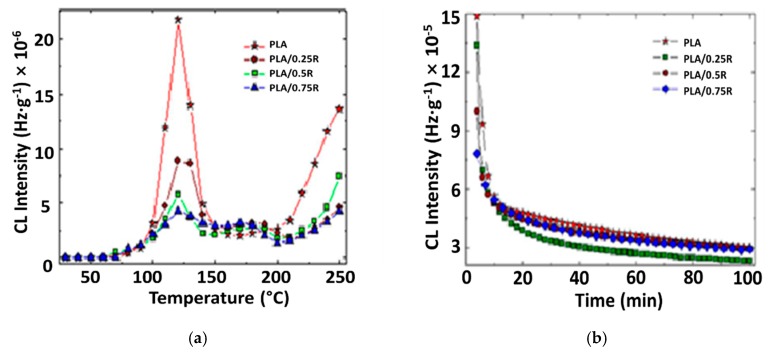
Chemiluminescence (CL) spectra recorded on PLA-based samples irradiated at 20 kGy: (**a**) nonisothermal measurements, heating rate: 3.7 °C min^−1^; (**b**) isothermal measurements: testing temperature 180 °C

The addition of natural phenolic antioxidants like powdered rosemary ethanolic extract increases the thermoxidative and radiation stabilities by scavenging degradation precursors. The contribution revealed by the components of this natural stabilizing mixture is observed by means of the differences between the CL intensities recorded for stabilized systems. The sharp progress in the radiation-stimulated degradation of PLA, see Figure 13a, is the result of the fast energy transfer from incidental radiation onto macromolecules followed by a high radiochemical yield of scission. In Figure 13b, the blocking action of oxidation is demonstrated by the diminution in the evolution of emission intensities, which drops as a parabolic decrease in the non-irradiated sample and the sudden hindrance of oxidation in the irradiated PLA/rosemary specimens.

The comparison between progress in the oxidation of irradiated neat and stabilized PLA emphasizes that the scavenging action of free radicals is selective because the deceleration of oxidation does not happen similarly. The chemiluminescence intensity is gradually diminished over time in protected polymer samples, while the radicals are suddenly oxidized in pristine PLA.

**Figure 14:**
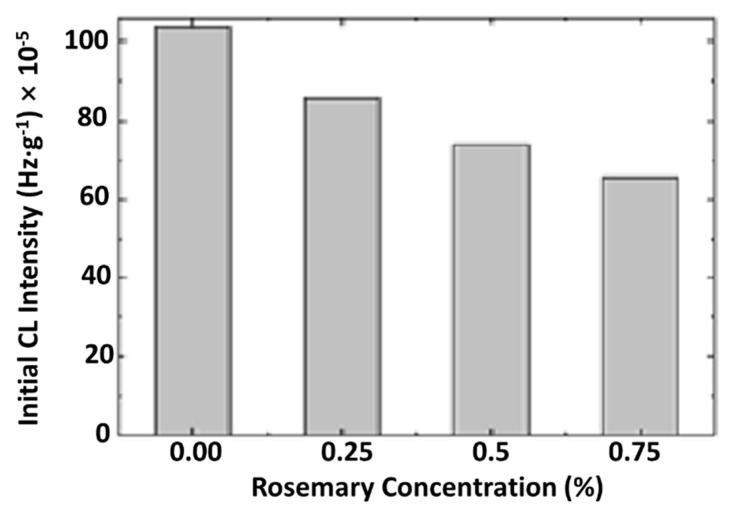
The dependence of the CL intensity on the concentration of powdered rosemary ethanolic extract.

The improvement in the thermal resistance of PLA in the presence of R is revealed in Figure 14. The linear modification of this parameter sustains the proportionality between the increase in the protection activity and material loading with rosemary ethanolic extract.

The modification in the rosemary loading can provide a remarkable effect on stabilization. If the concentrations of 0.25% and 0.50% bring small contributions at medium oxidation temperatures, the degradation is effectively delayed at temperatures exceeding 170 °C. The concentration of 0.75% R, delivers a significant effect toward the preservation of the oxidation state in PLA samples. The low CL intensities recorded for these last-mentioned samples in comparison with the low-loaded material is evidence for the involvement of the powder component in the breaking action on the oxidation chain process.

The stabilization profiles for PLA mixed with PEG in the presence of rosemary are different if the concentrations of radicals are changed. As the literature data mentioned, “the components of rosemary extracts are very efficient stabilizers against oxidation by blocking free radicals towards reaction with oxygen according to the mechanism proposed by Pospišil” [72]. Thus, a cascade mechanism is responsible for the oxidation of carnosic acid, forming other intermediates with active antioxidant structures showing a high level in stabilization activity. The stabilization effectiveness depends on the active phenolic structures and the flavonoid components contained in rosemary extract [27,73].

The initial intensity of CL emission is related to the concentration of peroxy species [74]. A value of 1.63 × 10^−3^ Hz/g was found for the neat PLA/PEG system, while that of PLA/PEG/0.5R was 0.18 × 10^−3^ Hz/g, about nine times lower than that of the system without R, indicating a good oxidative stabilization of the system is achieved through this additive.

It can be concluded, that the non-isothermal oxidation starts at high temperatures, decreasing with R content. The isothermal CL determinations point out an evident contribution of powdered rosemary ethanolic extract to the polymer stability and the retardation of oxidation to long exposure times.

### 3.9. Antioxidant Activity Evaluation

The radical scavenging activity of the powdered rosemary ethanolic extract was determined by ABTS*•^+^* method, and an IC50 (the concentration of the sample required to inhibit 50% of radicals) of 26 µg/mL was obtained, which proved that the powdered rosemary ethanolic extract has very good antioxidant activity.

**Figure 15:**
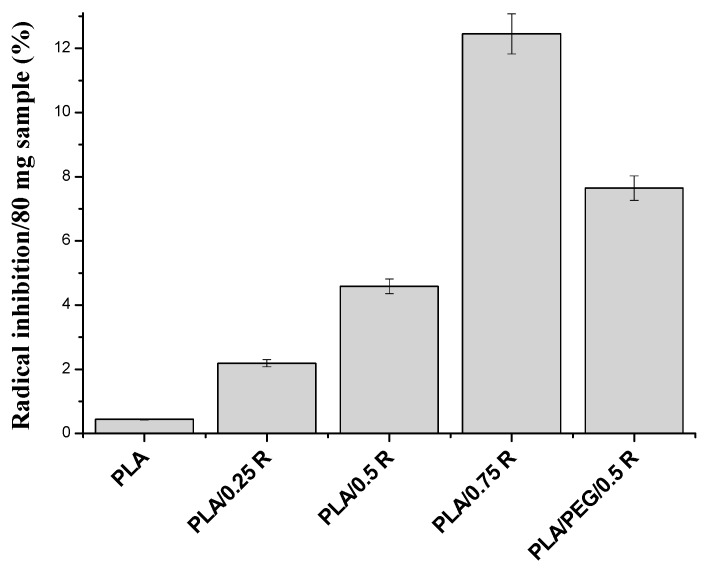
ABTS*•^+^* radical inhibition activity of the polymeric blends containing powdered rosemary ethanolic extract.

In Figure 15, the ABTS*•^+^* radical scavenging activity of PLA blends with different contents of powdered rosemary ethanolic extract is presented. The free radical scavenging activity in polymeric systems is directly proportional with the content of powdered rosemary ethanolic extract of the blend and is again higher for the plasticized PLA. The antioxidant activity of plasticized PLA/R samples is comparable to other systems based on plasticized PLA. For example, Lopusiewicz et al. [75] found an antioxidant activity value of 23% for PLA/melanin composites, and Byun et al. [76] determined an antioxidant activity of 90% and 14% for PLA-based blends with α-tocopherol or buthylated hydroxytoluene (BHT), respectively.

The obtained results are in accordance with those obtained by the chemiluminescence method.

### 3.10. Antibacterial Activity

*Escherichia coli* and *Salmonella typhimurium* are Gram-negative bacteria while *Bacillus cereus* or *B. cereus* is a Gram-positive, rod-shaped, aerobic, facultative anaerobic, motile, beta hemolytic bacterium commonly found in soil and food that produces toxins. These toxins can cause two types of illness: One type is characterized by diarrhea and the other, called emetic toxin, by nausea and vomiting. These bacteria can multiply quickly at room temperature [77]. Both Gram-negative bacteria and Gram-positive bacteria are susceptible to the antibacterial activity of powdered rosemary ethanolic extract, and their sensitivity is highly variable, see Table 7. The antimicrobial activity at 24 h increases with an increasing concentration of the powdered rosemary ethanolic extract, reaching values of about 100% for a concentration of 0.75%, and is higher in the case of the plasticized PLA systems because of better distribution of the powder into the matrix, as the SEM results demonstrated. It is worthwhile to mention the promising results obtained against *B*. *cereus*, as it is known that very serious problems are caused by this bacterium in association with food poisoning. Also, this bacterium has been associated with a multitude of other clinical conditions such as anthrax-like progressive pneumonia, severe eye infections, and devastating central nervous system infections, etc. Its role in nosocomial acquired bacteremia and wound infections in postsurgical patients has also been demonstrated, especially when intravascular devices such as catheters are inserted*.* *B*. *cereus* produces a potent β-lactamase conferring a marked resistance to β-lactam antibiotics [78].

The activity is lower in the case of *Salmonella typhymurium*. These results are in accordance with those found by other authors. Golshani and Sharifzadeh tested the rosemary alcoholic extract against *Staphylococcus aureus*, *Escherichia coli*, *Bacillus cereus*, and *Pseudomonas aeruginosa*, and proved its inhibitory effect for all these strains [79]. Some inhibitory effect for bacteria growth of PLA could be explained by the acidic pH imparted by it to the culture medium (pH 2), which is not favorable to bacteria growth and is due to the residual content of lactic acid.

### 3.11. Gas Permeability

It is well-known that the properties of PLA are compared to other commodity plastics, and the PLA permeation is shown to closely resemble that of polystyrene. Crystallinity was found to dominate permeation properties in a biaxially-oriented film [80]. The permeability depends on the film thickness and polymer morphology. Improvement of the gas permeability of the PLA-based films of approximately the same thickness in the presence of natural additive R is evident by comparing the data of Table 8.

The gas barrier improvement depends on the amount of R in the film’s composition both in PLA/R and plasticized PLA/R blends. The oxygen barrier properties are better than those of CO_2_. The results are in accordance with those obtained by Yuniarto et al*.* [81]. They found that the plasticized PLA by PEG enhanced the permeability value by about 20%, while the largest fraction PEG400 reduced the ability to prevent oxygen from passing through the film. In this case, the barrier properties were significantly affected by the degree of crystallinity in the film with a correlation number of 0.85. The gas permeability of two commercial food packages (a foil and a freezing bag) was also measured for comparative purposes. An unexpectedly high permeability for CO_2_ and for O_2_ was found for both packages. However, taking into consideration the difference in film thickness between our samples and the commercial ones, we do consider that PLA-based composites could be suitable for use as packages for food applications.

### 3.12. Overall Migration of Active Components from PLA/R Formulations into 50% Ethanol Solution as a Food Simulant Medium

**Figure 16:**
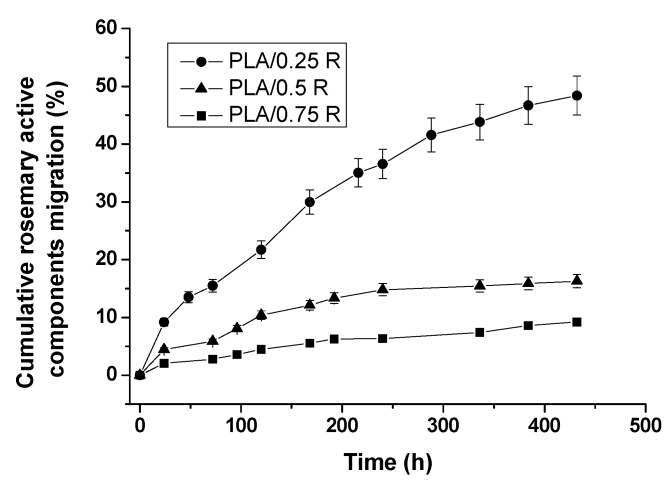
Migration profiles for the active ingredients of the powdered rosemary ethanolic extract (R) from PLA-based samples into 50% ethanol solution as a food simulant medium.

As mentioned in the experimental section, the overall migration of active components from PLA/R-based formulations into the simulant medium was determined according to EU regulations, and the results are summarized in Figure 16 and Table 9.

The migration profiles from PLA/R films with three different R compositions show a quite similar release behavior of the rosemary ethanolic extract active ingredients, but it is quantitatively influenced by the amount of R incorporated in the film samples. Thus, in the 18-day interval investigated, the active ingredients of R were released faster and in the highest amount from the PLA/0.25R sample—reaching 48.4% release, followed by the PLA/0.5R sample with 16.3% released, and the PLA/0.75R sample with only 9.2% released. The increase of the R content leads to a slower release of the active components.

The calculated kinetic parameters and the corresponding correlation coefficient values (R^2^) are summarized in Table 9.

The release exponent, *n* values, obtained by fitting the data to the Korsmeyer-Peppas equation, see Equation (2) [36], are 0.61 for PLA/0.25R, 0.42 for PLA/0.5R, and 0.55 for PLA/0.75R, all these values are situated around 0.5, indicating a behavior closer to Fickian diffusion, thus, in general, a diffusion-controlled mechanism characterizes the migration process of the bioactive components from the polymeric films. A decrease of the release rate constant values with an increasing amount of R incorporated (the lowest k = 3.52 × 10^−3^ for PLA/0.75R sample) was observed, which corresponds to a slower release, as shown by the migration profiles.

A good fit was obtained for the Higuchi model, see Equation (3) [37], as the R^2^ values suggest, indicating diffusion as the preferred mechanism for migration, with the same trend of decreasing k_H_ values: k_PLA/0.25R_ > k_PLA/0.5R_ > k_PLA/0.75R_ was found through the application of both models.

These results indicate that the migration rate of some components of the rosemary ethanolic extract from PLA/R films directly depends on their permeability and degree of crystallization. For films of approximately the same thickness, the gas permeability decreased by two or six times, indicating difficult diffusion of components through the materials. Also, the interaction between the PLA/R blends components can slow the migration into the food simulant.

### 3.13. Biocompatibility Evaluation

#### 3.13.1. In Vitro Biocompatibility Evaluation Based on Surface Properties

The contact angles decrease for all systems with respect to that of PLA, see Table 10, indicating that an increased hydrophilicity was achieved through R incorporation. This increase is also proved by variations recorded in the surface tension and its components. The total surface tension $\gamma_{sv}^{TOT}$ increases with R incorporation, and, as was the case for other properties, the highest values were registered for the PLA/PEG/0.5R system.

The most important change was found in the $\gamma_{sv}^{-}$ electron donor (Lewis base) component which is 0.19 mN/m for PLA and increases to 38.05 mN/m for PLA/PEG/0.5R while $\gamma_{sv}^{AB}$, polar Lewis acid-base interaction varied from 0.86 mN/m to 13.99 mN/m. This phenomenon is related by the cohesion between molecules at the surface of the liquid. It is known that the stronger the intermolecular interactions, the greater the surface tension [82]; therefore, rosemary extract improved the interactions of the liquids with surfaces, obtaining composites with better water wettability. The presence of PEG as a plasticizer into the polymeric system enhances the surface hydrophilicity; the lowest CA was recorded for PLA/PEG (50°) compared with that of PLA (84.7°), see Table 10. The decrease of CA is directly correlated with the content of rosemary ethanolic extract, indicating that by increasing the content of rosemary extract, more hydrophilic groups are accessible at the top surface, obtaining a material with a moderate surface wettability (64.4° for 0.75% content rosemary extract).

In Table 11, the values of the surface tension components (determined by acid/base method) and work of spreading for red blood cells and platelets are presented, indicating that with the addition of rosemary ethanolic extract and PEG into the PLA, the total SFE increases. This change of the surface free energy may lead to the improvement of the bio-adhesive ability of the material. In the interaction of biomaterials with tissues, their polar character has an important role, which is reflected in the SFE’s polar component ($\gamma_{sv}^{AB}$), see Table 11. At the surface of PLA, Lifshitz-van der Waals interactions prevail over acid-base interactions, by comparison with the surfaces of its composites. Blending PLA with rosemary ethanolic extract and PEG determines an increase in the contribution of the polar components to the total surface energy, having a more pronounced polar character than PLA, which is indicated by the higher value of $\gamma_{sv}^{AB}$ component. The basic component ($\gamma_{sv}^{-}$) of the surface free energy is much higher than the acidic component ($\gamma_{sv}^{+}$), suggesting that the surface of the composites is mainly monopolar with a Lewis base character, due to the surface being enriched with electron donor functional groups (–OH, O–C=O) from rosemary ethanolic extract. If polymeric materials are used as implant biomaterials, strong cell adhesion and rapid cell growth on the polymer surface will generally be beneficial for the incorporation of the device into the body. Van der Valk et al*.* [83] have shown that cell (fibroblast) spreading appeared to be dependent on the polar surface free energy. Cell spreading is low when the SFE’s polar part of the material is lower than 5 mN/m, and marked spreading occurs when $\gamma_{sv}^{AB}$ is higher than 15 mN/m [43]. Based on the values of the SFE polar part determined for the PLA/R composites, it can be assumed that the sample incorporating all the components (PLA/PEG/0.5R) will give better spreading and division of the fibroblasts because their $\gamma_{sv}^{AB}$ takes an intermediary value.

When blood comes into contact with a biomaterial, the surface properties, especially its wettability, play a crucial role. The response of platelets towards the hydrophobic or hydrophilic materials is different [84]. In the presence of a biomaterial, platelets’ adherence to the foreign material and activation, usually results in the initiation of platelet aggregation, further initiating thrombosis by secreting prothrombotic factors that lead to clotting [85]. Under normal conditions, thrombosis and complement activation are favorable responses which prevent blood loss. However, in the presence of a foreign material such as a blood-contacting device, thrombosis, and complement activation are unfavorable, leading to blood clots and hence a low biocompatibility [84]. Thus, a blood-contacting device that does not elicit thrombosis would be considered biocompatible [86]. As revealed in Table 11, the work of spreading of the red blood cells, *W_s/rbc_*, has positive values (except for the PLA and PLA/0.5R samples) and the work of the spreading of platelets, *W_s/p_*, has negative values, which suggests that the work of adhesion is higher than that of cohesion for the red blood cells, but, comparatively, a smaller work of adhesion than that of cohesion for platelets. These findings denote that the contact of blood with PLA/R-based composites causes an increase in the work of cohesion for platelets; hence the platelets will not adhere easily onto the biomaterial’s surface, thus avoiding the appearance of thrombosis [87].

#### 3.13.2. In Vivo Biocompatibility Evaluation

No significant modifications between the percentage values of blood leucocyte formula elements were discovered in rats with PLA/0.25R, PLA/0.5R, PLA/0.75R, PLA/PEG, and PLA/PEG/0.5R implants and those of control group, PLA, and R, respectively, at 24 h and 7 days in the experiment, see Table 12.

Laboratory investigations did not reveal substantial variations of AST, ALT, and LDH activity between groups with the PLA-based materials containing rosemary ethanolic extract pellets and the control, PLA, and R groups, respectively, 24 h and 7 days after the implantation, see Table 13.

The implantation of pellets with PLA/0.25R, PLA/0.5R, PLA/0.75R, PLA/PEG and PLA/PEG/0.5R, did not induce significant differences in the serum levels of urea and creatinine compared with the groups receiving PLA, R, and the control, see Table 14.

No major dissimilarities were observed in the values of serum complement and the phagocytic capacity of peripheral neutrophils between the groups with PLA-based materials containing rosemary ethanolic extract implants, and PLA, R, and the control group, respectively, at 24 h and 7 days after the implantation see Table 15.

All these results indicate a good and increased biocompatibility of the PLA/R which recommends them as biomaterials for various applications because rosemary ethanolic extract offers both an improved biocompatibility of PLA-based materials and it also confers the different biological activities mentioned above.

## 4. Conclusions

New multifunctional materials based on PLA-containing additives derived from natural resources were obtained by melt mixing. 

Incorporation of powdered rosemary ethanolic extract into PLA improved the elongation at break, rheological and thermal properties, and antibacterial and antioxidant activities. Additionally, the novel materials showed a good compatibility and in vitro and in vivo biocompatibility. The contribution of additive to the protection of PLA against oxidative degradation is discussed with respect to the phenolic compounds contained in rosemary powder.

A good agreement between results was found, such as between those of chemiluminescence and radicals’ scavenging activity determination via a chemical method that evidenced the increased thermoxidative stability of the PLA biocomposites containing powdered rosemary ethanolic extract which acts as an antioxidant. These biocomposites show low migration rates of the bioactive compounds from matrices and permeability to gases, and, therefore, can be considered as high-performance materials for food packaging.

In vitro biocompatibility based on the determination of surface properties demonstrated a good hydrophilicity, better spreading and division of fibroblasts, and an increase of platelets’ cohesion. Therefore, they will not adhere easily on the biomaterial’s surface thus avoiding the appearance of thrombosis.

It was demonstrated that implantation of PLA-based materials containing rosemary ethanolic extract resulted in similar blood parameter changes and biochemical responses with the control group, the group treated with pellets impregnated with R solution, and with PLA pellets, respectively.

The subcutaneous application of PLA-based materials containing rosemary ethanolic extract pellets did not significantly influence the immune reactivity of rats, compared with PLA, R, and the control group.

It can be concluded that, in our laboratory conditions, the implantation of PLA/0.25R, PLA/0.5R, PLA/0.75R, PLA/PEG, and PLA/PEG/0.5R pellets, proved a good in vivo biocompatibility, suggesting that these PLA-based materials containing rosemary ethanolic extract show very good properties as potential biomaterials, which could be further used for evaluating their possible pharmacodynamics effects in different experimental models in animals.

The PLA/R-based materials show promising properties for application both in biodegradable food packaging and as biomaterials with bioactive activities.

## Figures and Tables

**Table 1:** Compositions of the prepared poly(lactic acid) (PLA)-based systems.

No.	Sample	PLA (wt %)	Powdered Rosemary Ethanolic Extract (R) (wt %)	PEG (wt %)
1	PLA	100	-	-
2	PLA/0.25R	99.75	0.25	-
3	PLA/0.5R	99.5	0.5	-
4	PLA/0.75R	99.25	0.75	-
5	PLA/PEG	80	-	20
6	PLA/PEG/0.5R	79.5	0.5	20

**Table 2:** Surface tension parameters (mN/m) of the liquids used for contact angle measurements.

Liquid	$\gamma_{lv}^{TOT}$	$\gamma_{lv}^{LW}$	$\gamma_{lv}^{AB}$	$\gamma_{lv}^{+}$	$\gamma_{lv}^{-}$
Water	72.80	21.80	51.00	25.50	25.50
Formamide	58.00	39.00	19.00	2.28	39.6
Methylene iodide	50.80	50.80	0.00	0.72	0.00
Red blood cells (rbc) [49]	36.56	35.2	1.36	0.01	46.2
Platelets (p) [49]	118.24	99.14	19.1	12.26	7.44

**Table 3:** Melt processing characteristics of PLA and its blends with rosemary solid extract.

Sample	TQ_max1_ (Nm)	TQ_1min_ (Nm)	TQ_max2_ (Nm)	TQ_5min_ (Nm)	TQ_final_ (Nm)
PLA	66.4	17.2	-	13.3	10.2
PLA/0.25R	73.8	15.7	-	11.2	10.2
PLA/0.5R	72.0	17	-	11.4	10.5
PLA/0.75R	74.6	17.6	-	11.1	10.1
PLA/PEG	12.9	0.9	-	7.3	6.5
PLA/PEG/0.5R	10.1	2.8	-	7.3	5.7

TQ_max1_—maximum torque; TQ_1min_—torque after one minute of mixing; TQ_max2_—maximum torque after 1.5 min of mixing; TQ_5min_—torque after 5 min of mixing (half processing time); TQ_final_—torque at the end of mixing.

**Table 4:** Crossover frequency and crossover moduli values for PLA, and PLA/Rosemary blends.

Crossover Characteristic	PLA	PLA/0.25R	PLA/0.5R	PLA/0.75R
ω (s^−1^)	96.37	273.3	363.3	411.4
*t* (s)	10.38	3.66	2.75	2.43
*G’* = *G"* (MPa)	144.5	146.9	145.2	145.4

**Table 5:** Thermal characteristics of the PLA/R and plasticized PLA/R systems determined by DSC method.

Sample	*T_g_* (°C)	*T_cc_* (°C)	*∆H_cc_* (J/g)	*T_m_* (°C)	∆*H_m_* (J/g)	*T_cr_* (°C)	*X_cr_*
Run I							
PLA	60.64	125.89	17.69	151.63	23.12	55.80	24.6
PLA/0.25R	62.95	124.56	18.23	155.96	19.66	55.96	21.1
PLA/0.5R	64.07	124.82	10.40	154.51	10.78	56.26	11.5
PLA/0.75R	64.82	118.28	16.54	156.35	19.05	55.86	20.33
PLA/PEG	49.30	-	-	157.74	23.95	70.03	25.56
PLA/PEG/0.5R	59.59	90.23	2.6	157.07	26.06	66.70	28.39
Run II							
PLA	61.58	132.03	9.69	152.75	12.05	-	13.34
PLA/0.25R	60.96	128.95	17.14	154.01	16.4	-	17.50
PLA/0.5R	61.15	133.87	6.24	153.90	6.37	-	6.79
PLA/0.75R	60.40	133.48	7.18	154.04	7.9	-	8.43
PLA/PEG	-	91.37	0.36	154.66	25.32	-	27.02
PLA/PEG/0.5R	-	80.93	8.003	153.72	27.28	-	29.11

*T_g_*—glass transition temperature; *T_cc_*—cold crystallization temperature; *T_m_*—melting temperature; *T_cr_*—crystallization temperature; *ΔH_cc_*—cold crystallization enthalpy; Δ*H_m_*—melting enthalpy; *X_cr_*—crystallinity index.

**Table 6:** TG data for PLA containing powdered rosemary ethanolic extract.

Sample	Degradation Stage	*T_onset_* (°C)	*T_peak_* (°C)	Δ*W* (%)	*T*_10_ (°C)	*T*_20_ (°C)
PLA	I residue	339	358	98.72 1.28	332.5	341.5
PLA/0.75R	I residue	310	338	98.50 1.50	312.5	321.5
PLA/PEG	I II residue	280 373	322 402	82.51 16.31 1.18	285.5	298
PLA/PEG/0.5R	I II residue	285 365	336 403	83.03 15.04 1.93	294	207.5

*T_onset_*—the temperature at which the thermal degradation start; *T_peak_*—the temperature at which the degradation rate is maximum; *T*_10_, *T*_20_—the temperatures corresponding to 10 wt % and 20 wt % mass losses; Δ*W*—residual mass at 600 °C*.*

**Table 7:** Antibacterial activity of the alcoholic extract from rosemary incorporated into PLA-based materials against *Bacilus cereus*, *Salmonella typhymurium*, and *Escherichia coli*.

Sample	ATCC *Bacillus cereus* 14579		ATCC *Salmonella typhymurium* 14028		ATCC *Escherichia coli* 25922	
	Inhibition %/24 h	Inhibition %/48 h	Inhibition %/24 h	Inhibition %/48 h	Inhibition %/24 h	Inhibition %/48 h
PLA	5	59	32	61	53	71
PLA/0.25 R	59	100	52	87	61	86
PLA/0.5 R	91	100	52	84	71	100
PLA/0.75 R	100	100	55	87	94	100
PLA/PEG	45	91	29	77	69	94
PLA/PEG/0.5R	86	100	48	100	76	100

**Table 8:** Permeability of tested films to CO_2_ (~4 h to equilibrium) and O_2_ (~7 h to equilibrium).

Sample	Thickness (mm)	CO_2_ (mL/m^2^/day)	O_2_ (mL/m^2^/day)
PLA	0.151	873	1308
PLA/0.25R	0.120	588	487
PLA/0.5R	0.122	535	273
PLA/0.75R	0.130	412	201
PLA/PEG	0.128	524	455
PLA/PEG/0.5R	0.126	489	278
Food freezing bag	0.020	64,601	50,266
Food packaging foil (LDPE/PP)	0.009	128,374	35,629

**Table 9:** Migration kinetic parameters for the active ingredients of the powdered rosemary ethanol extract from PLA-based materials into 50% ethanol solution as a food simulant medium.

Sample	Korsmeyer-Peppas Model				Higuchi Model	
	*n*	R^2^	K × 10^−3^ (h)^−n^	R^2^	k_H_ × 10^−3^ (h)^−n^	R^2^
PLA/0.25R	0.61	0.99	14.1	0.989	27.57	0.98
PLA/0.5R	0.42	0.98	12.82	0.988	8.28	0.98
PLA/0.75R	0.54	0.985	3.52	0.993	4.48	0.99

**Table 10:** The contact angle values (*Ɵ*) between liquids (water, formamide, or diiodomethane) and surfaces of the sample of different compositions.

Samples	Contact Angles Values (degrees)		
	Water	Formamide	Diiodomethane
PLA	84.7	70.9	65.0
PLA/0.25R	81.9	62.7	60.1
PLA/0.5R	78.8	59.0	56.7
PLA/0.75R	64.4	42.8	38.7
PLA/PEG	50.0	46.4	40.7
PLA/PEG/0.5R	63.7	71.0	51.0

**Table 11:** Surface tension parameters (mN/m) and work of spreading for red blood cells and platelets of composites samples.

Samples	$\gamma_{sv}^{LW}$	$\gamma_{sv}^{AB}$	$\gamma_{sv}^{+}$	$\gamma_{sv}^{-}$	$\gamma_{sv}^{TOT}$	*W_s/rbc_*	*W_s/p_*
PLA	25.65	1.75	0.06	12.45	27.40	−8.95	−109.56
PLA/0.25R	28.46	3.31	0.62	7.22	32.69	1.43	−107.13
PLA/0.5R	30.41	4.23	0.11	24.01	33.72	−2.11	−90.51
PLA/0.75R	40.17	5.41	0.33	22.22	45.58	10.83	−74.12
PLA/PEG	39.17	1.92	0.03	34.91	41.09	4.54	−69.58
PLA/PEG/0.5R	33.64	13.95	1.27	38.25	47.59	12.27	−71.51

*θ* = contact angle; $\gamma_{sv}^{TOT}$ = liquid’s total surface tension; $\gamma_{lv}^{LW}$ and $\gamma_{sv}^{LW}$ = apolar Lifshitz—van der Waals components of the liquid and the solid $\gamma_{sv}^{+}\gamma_{lv}^{-}$γ_lv_^-^ and $\gamma_{sv}^{-}\gamma_{lv}^{+}$ = Lewis acid-base contributions of either the solid or the liquid phase; $\gamma_{\mathrm{sv}}^{-}$= electron donor (Lewis base) and $\gamma_{sv}^{+}$ = electron acceptor (Lewis acid) components; “*lv*” and “*sv*” = denote the interfacial liquid-vapor and surface-vapor tensions; “p” and “d” = denote the polar and disperse components of total surface tension, $\gamma_{lv}^{TOT}$;$\gamma_{sv}^{AB}$ = polar Lewis acid-base interaction.

**Table 12:** The effects of the PLA-based materials containing rosemary ethanolic extract on the differential white cell count. Values were expressed as mean ± S.D. for six rats in a group.

Group		Leucocyte Formula				
		% Values				
		PMN	Ly	E	M	B
Control	24 h	29.5 ± 0.83	66.3 ± 2.11	0.6 ± 0.08	3.4 ± 0.10	0.2 ± 0.10
	7 days	29.7 ± 0.47	65.9 ± 1.93	0.7 ± 0.10	3.5 ± 0.10	0.2 ± 0.05
R	24 h	29.5 ± 0.69	66.1 ± 1.75	0.8 ± 0.06	3.4 ± 0.06	0.2 ± 0.10
	7 days	29.6 ± 0.73	66.2 ± 1.89	0.6 ± 0.08	3.4 ± 0.10	0.2 ± 0.05
PLA	24 h	29.6 ± 0.89	66.1 ± 2.13	0.6 ± 0.05	3.5 ± 0.05	0.2 ± 0.05
	7 days	29.7 ± 1.13	65.9 ± 1.55	0.7 ± 0.05	3.5 ± 0.05	0.2 ± 0.04
PLA/0.25R	24 h	29.6 ± 0.21	66.0 ± 1.73	0.7 ± 0.12	3.5 ± 0.08	0.2 ± 0.04
	7 days	29.8 ± 1.13	65.7 ± 1.29	0.7 ± 0.05	3.6 ± 0.10	0.2 ± 0.05
PLA/0.5R	24 h	29.7 ± 0.29	65.9 ± 2.14	0.6 ± 0.10	3.6 ± 0.08	0.2 ± 0.10
	7 days	29.7 ± 1.17	65.7 ± 1.33	0.8 ± 0.06	3.6 ± 0.05	0.2 ± 0.05
PLA/0.75R	24 h	29.6 ± 0.98	65.8 ± 1.67	0.8 ± 0.12	3.6 ± 0.10	0.2 ± 0.10
	7 days	29.8 ± 0.73	65.8 ± 1.75	0.6 ± 0.05	3.6 ± 0.08	0.2 ± 0.05
PLA/PEG	24 h	29.8 ± 0.89	65.7 ± 1.39	0.7 ± 0.10	3.6 ± 0.05	0.2 ± 0.04
	7 days	29.9 ± 0.55	65.5 ± 1.63	0.7 ± 0.05	3.7 ± 0.12	0.2 ± 0.05
PLA/PEG/0.5R	24 h	29.8 ± 0.27	65.7 ± 1.98	0.6 ± 0.10	3.7 ± 0.10	0.2 ± 0.05
	7 days	29.9 ± 1.63	65.4 ± 1.47	0.8 ± 0.13	3.7 ± 0.05	0.2 ± 0.05

PMN*—*polymorphonuclear leukocytes; Ly*—*lymphocytes; E—eosinophils; M—monocytes; B—basophils.

**Table 13:** The effects of PLA-based materials containing rosemary ethanolic extract on the activity of AST, ALT, and LDH. Values were expressed as mean ± S.D. for six rats in a group.

Group		AST (U/mL)	ALT (U/mL)	LDH (U/mL)
Control	24 h	41.7 ± 2.72	95.3 ± 4.14	342.29 ± 44.55
	7 days	42.5 ± 3.07	96.5 ± 3.89	344.33 ± 41.37
PLA	24 h	42.3 ± 3.14	95.8 ± 4.46	342.17 ± 39.64
	7 days	42.9 ± 2.33	97.6 ± 3.55	345.25 ± 40.89
R	24 h	41.6 ± 1.89	96.2 ± 3.37	343.42 ± 42.14
	7 days	42.7 ± 2.64	98.7 ± 5.07	346.67 ± 38.33
PLA/0.25R	24 h	42.1 ± 3.14	97.5 ± 4.27	343.54 ± 43.46
	7 days	43.9 ± 3.46	97.9 ± 3.64	346.81 ± 41.37
PLA/0.5R	24 h	43.2 ± 3.33	97.6 ± 3.37	344.55 ± 39.89
	7 days	44.1 ± 3.27	98.8 ± 5.14	347.19 ± 44.14
PLA/0.75R	24 h	43.4 ± 2.37	98.3 ± 5.55	344.29 ± 43.72
	7 days	44.6 ± 2.55	98.7 ± 6.07	348.46 ± 45.07
PLA/PEG	24 h	43.7 ± 3.37	98.5 ± 5.46	345.72 ± 44.37
	7 days	44.8 ± 3.89	98.8 ± 4.33	349.15 ± 40.46
PLA/PEG/0.5R	24 h	43.5 ± 3.14	97.2 ± 3.89	345.45 ± 39.89
	7 days	44.6 ± 3.64	98.6 ± 5.72	347.83 ± 42.27

AST—aspartate transaminase, ALT—alanine aminotransferase and LDH—lactic dehydrogenase*.*

**Table 14:** The effects of PLA-based materials containing rosemary ethanolic extract on the serum urea and creatinine concentration. Values were expressed as mean ± S.D. for six rats in a group.

Group		Urea (mg/dL)	Creatinine (mg/dL)
Control	24 h	37.2 ± 3.37	<0.1
	7 days	37.9 ± 4.55	<0.1
PLA	24 h	37.7 ± 3.89	<0.2
	7 days	38.1 ± 3.64	<0.2
R	24 h	37.9 ± 5.07	<0.1
	7 days	38.5 ± 3.46	<0.1
PLA/0.25R	24 h	38.6 ± 4.37	<0.1
	7 days	38.9 ± 4.33	<0.2
PLA/0.5R	24 h	38.8 ± 3.64	<0.2
	7 days	39.3 ± 3.72	<0.2
PLA/0.75R	24 h	39.1 ± 5.14	<0.2
	7 days	39.4 ± 4.46	<0.2
PLA/PEG	24 h	38.8 ± 3.55	<0.2
	7 days	39.6 ± 3.27	<0.2
PLA/PEG/0.5R	24 h	39.2 ± 4.64	<0.2
	7 days	39.5 ± 3.37	<0.2

**Table 15:** The effects of PLA-based materials containing rosemary ethanolic extract on the serum complement activity and the NBT test. Values were expressed as mean ± S.D. for six rats in a group.

Group		Complement	NBT Test
Control	24 h	16.33 ± 1.55	53.73 ± 3.46
	7 days	16.48 ± 1.37	53.65 ± 3.55
PLA	24 h	16.41 ± 1.46	53.85 ± 4.14
	7 days	16.65 ± 1.14	53.49 ± 3.72
R	24 h	16.39 ± 1.55	52.63 ± 3.50
	7 days	16.47 ± 1.33	52.45 ± 3.14
PLA/0.25R	24 h	16.43 ± 1.64	52.77 ± 3.67
	7 days	16.85 ± 0.89	52.39 ± 3.25
PLA/0.5R	24 h	16.56 ± 1.72	53.68 ± 3.46
	7 days	16.89 ± 1.37	52.55 ± 3.83
PLA/0.75R	24 h	16.77 ± 1.64	53.73 ± 3.46
	7 days	17.11 ± 0.72	52.61 ± 3.37
PLA/PEG	24 h	17.07 ± 0.89	53.85 ± 4.05
	7 days	16.63 ± 1.55	53.37 ± 3.64
PLA/PEG/0.5R	24 h	17.03 ± 0.83	54.19 ± 4.17
	7 days	17.13 ± 1.46	53.49 ± 3.55
